# EFFECTS OF HAMMOCK POSITIONING ON CLINICAL PARAMETERS IN PRETERM INFANTS ADMITTED TO A NEONATAL INTENSIVE CARE UNIT: A SYSTEMATIC REVIEW

**DOI:** 10.1590/1984-0462/2021/39/2019399

**Published:** 2020-11-30

**Authors:** Janaina de Lima Menger, Letícia Reck Mafaldo, Daniele Schiwe, Camila Wohlgemuth Schaan, João Paulo Heinzmann-Filho

**Affiliations:** a Universidade Federal do Rio Grande do Sul, Porto Alegre, RS, Brazil.; b Hospital Moinho de Ventos, Porto Alegre, RS, Brazil.; c Pontifícia Universidade Católica do Rio Grande do Sul, Porto Alegre, RS, Brazil.; d Hospital de Clínica de Porto Alegre, Porto Alegre, RS, Brazil.; e Centro Universitário Cenecista de Osório, Osório, RS, Brazil.

**Keywords:** Premature newborn, Patient positioning, Neonatal Intensive Care Units, Recém-nascido prematuro, Posicionamento do paciente, Unidades de Terapia Intensiva Neonatal

## Abstract

**Objective:**

To review the effects of the hammock positioning on clinical parameters of preterm newborn infants (PTNB) admitted to the Neonatal Intensive Care Unit (NICU).

**Data sources:**

This was a systematic review performed by searching the Pubmed, Lilacs, SciELO and PEDro databases. Intervention studies in English, Portuguese and Spanish that evaluated the effects of hammock positioning on clinical parameters of PTNB admitted to the NICU were selected. Three search strategies were used: 1) hammock positioning OR patient positioning AND intensive care units AND infant, newborn; 2) hammock positioning OR patient positioning AND intensive care units; 3) hammock positioning OR patient positioning AND intensive care units, neonatal. There was no restriction on the year of publication of the articles. Methodological quality was assessed by the PEDro scale.

**Data synthesis:**

Among 597 articles, only six were included and 139 neonates with gestational ages between 26 and 37 weeks and an average gestational weight <2240g were analyzed. Four studies included patients without any associated pathology and most of them placed the PTNB supine in hammock positioning. The duration of the intervention ranged from 15 to 180 minutes and most applied it at just one moment. There was an improvement in heart rate (HR), respiratory rate (RR) and pain (3/4 studies), as well as gains in peripheral oxygen saturation (SpO2) (2/4 studies). Only one study reported worsening of SpO2 with the intervention. The methodological quality of the studies was classified as low.

**Conclusions:**

Although this review suggests improvement with hammock positioning in HR, RR and pain in PTNB, the low methodological quality makes the results inconsistent.

## RESUMO

**Objetivo:**

Revisar os efeitos da rede de posicionamento nos parâmetros clínicos de recém-nascidos pré-termo (RNPT) admitidos em Unidade de Terapia Intensiva Neonatal (UTIN).

**Fontes de dados:**

Trata-se de uma revisão sistemática realizada na PubMed, na Literatura Latino-Americana e do Caribe em Ciências da Saúde (Lilacs), na Biblioteca Eletrônica Científica Online (SciELO) e na Base de Dados em Evidências em Fisioterapia (PEDro). Selecionaram-se estudos de intervenção, nos idiomas inglês, português e espanhol, que avaliaram os efeitos da rede de posicionamento sobre parâmetros clínicos de RNPT admitidos em UTIN. Foram utilizadas três estratégias de busca: *hammock positioning OR patient positioning AND intensive care units AND infant, newborn*; *hammock positioning OR patient positioning AND intensive care units*; e *hammock positioning OR patient positioning AND intensive care units, neonatal.* Não houve restrição quanto ao ano de publicação dos artigos. A qualidade metodológica foi avaliada pela escala PEDro.

**Síntese dos dados:**

De um total de 597 artigos, apenas seis foram incluídos. As amostras totalizaram 139 neonatos, com idade gestacional entre 26 e 37 semanas e média de peso gestacional <2.240 g. Quatro estudos incluíram pacientes sem nenhuma patologia associada, e a maior parte deles dispôs os RNPT em supino na rede de posicionamento. A duração da intervenção variou de 15 até 180 minutos, e a maioria aplicou essa intervenção em apenas um momento. Foi observada melhora na frequência cardíaca (FC), na frequência respiratória (FR) e na dor (3/4 estudos), além de ganhos na saturação periférica de oxigênio (SpO_2_) (2/4 estudos). Apenas um artigo relatou piora da SpO_2_ com a intervenção. A qualidade metodológica foi classificada como baixa.

**Conclusões:**

Embora a rede de posicionamento pareça causar melhora na FC, na FR e na dor em RNPT, a baixa qualidade metodológica torna inconsistentes os resultados.

## INTRODUCTION

The synchronous-active theory of newborn neurobehavioral organization, proposed by Heidelise Als in 1982, describes newborns' behavioral organization and their development regarding the balance between children’s interaction with the environment and their neurobehavioral subsystems. The subsystems include the autonomous system, the motor system, behavioral status, attention-interaction and the regulatory system, all of which have a sequential and interdependent maturation.^1^ ^,^ ^2^

Preterm newborns (PTNB) admitted to the Neonatal Intensive Care Unit (NICU) may present changes in their neurobehavioral organization, as they are exposed to light, noise, handling and painful interventions. This is capable of causing physiological disorganization, energy expenditure, hemodynamic instability, changes in intracranial pressure and central nervous system involvement.^3^ ^,^ ^4^ ^,^ ^5^ ^,^ ^6^ ^,^ ^7^ Behavioral strategies for positioning and sensory stimulation are necessary in the NICU in order to minimize the losses triggered by these stressors and to promote comfort.^4^ ^,^ ^7^

Over the past 15 years, initiatives to humanize care within the NICU have emphasized the importance of this type of strategy, articulating the technical quality of care, welcoming technologies, support for patients and family members. These initiatives have been presented in several fields, but have been implemented *a priori* in care for childbirth and newborns. Among these actions, humanized birth, the kangaroo method, water immersion, music therapy and hammock positioning stand out.^8^ ^,^ ^9^ ^,^ ^10^ ^,^ ^11^

Hammock positioning is a method that is considered to be simple and low cost, consisting of positioning the PTNB in a hammock commonly made of fabric that has a rectangular shape and is fixed at the ends of the incubators.^9^ The therapeutic position with this method potentially simulates the intrauterine environment, providing relaxation and the development of spontaneous and functional motor skills, in addition to minimizing postural abnormalities and asymmetries related to prematurity and NICU stay.^4^ ^,^ ^7^ ^,^ ^12^ ^,^ ^13^ In Brazil, it is used mainly in the Northeast Region and, despite its applicability, there is little evidence regarding its indication. Hammock positioning has been studied in preterm infants and hemodynamically stable term infants who have not needed oxygen therapy.^8^ ^,^ ^14^

Bottos et al. in 1985,^15^ were the first researchers to compare cardiorespiratory outcomes in PTNB and those born at term with the use of hammock positioning or in the supine position. All research subjects were placed in incubators for 23 minutes, alternating in both positions. In this study, no significant changes were found in peripheral oxygen saturation (SpO_2_) among patients undergoing both strategies, even when stratifying them with regard to birth weight (≥2,000 g) and gestational age (≥35 weeks ).^15^ Over time, other studies have emerged, and in some of them, improvement has been observed in sleep,^4^ in relaxation,^16^ in neuropsychomotor development,^17^ in decreasing energy expenditure^18^ and in stress.^19^ Furthermore, a recent survey^7^ showed that the weight of PTNB in NICUs in hammock positioning was higher at the time of hospital discharge compared to those who received the kangaroo method.

Therefore, taking into account the frequent stressors of PTNB in the NICU and the different strategies of humanized care, especially simple management such as hammock positioning,^20^ the investigation into the possible effects of this method is justified. Furthermore, to date, no critical or systematic review has been found on the subject. Thus, the aim of the present study was to systematically review the effects of hammock positioning on clinical parameters of PTNB admitted to the NICU.

## METHOD

The systematic review was carried out in accordance with the recommendations of the Preferred Reporting Items for Systematic Reviews and Meta-Analyzes^21^ and by searching the PubMed databases via the Medical Literature Analysis and Retrieval System Online (MEDLINE), Latin American and Caribbean Literature on Health Sciences (LILACS), Scientific Electronic Library Online (SciELO) and the Physiotherapy Evidence Database (PEDro).

Intervention studies (clinical and/or quasi-experimental) were selected in English, Portuguese and Spanish, with no filter as to the age and year of publication of the articles. The study selection period was between May and September 2019.

The search used to select the articles was based on six keywords that were associated with Boolean descriptors. Three search strategies were used in each database:

- Hammock positioning OR patient positioning AND intensive care units AND infant, newborn.
- Hammock positioning OR patient positioning AND intensive care units.
- Hammock positioning OR patient positioning AND intensive care units, neonatal.

All of these terms are controlled descriptors, registered in the Health Sciences Descriptors (*Descritores em Ciências da Saúde* - DeCS), with the exception of the keyword “hammock positioning”. We chose to leave it because many studies use this term in abstracts. All descriptors had to have at least the title, abstract or keywords. In addition, the references of the included studies were reviewed to verify possible articles to compose the present work (grey literature)*.*

Studies that evaluated the effects of hammock positioning on clinical parameters (vital signs, pain, stress, sleep/wake, temperature, organizational status, neuromuscular maturity, autonomic stability and posture) of PTNB (<37 weeks), with/without associated pathologies and admitted to the NICU. On the other hand, abstracts, dissertations, theses, clinical guidelines, editorial letters, review articles, case reports, expert opinions and studies involving infants in their samples were excluded.

After identifying the descriptors in the title, in the abstract and/or in the keywords, the abstracts were read from the selected articles in order to assess adequacy regarding the eligibility criteria. The studies that presented the predetermined criteria had their full text acquired for detailed analysis and data extraction. The search and analysis of the articles were conducted independently by two reviewers, with any disagreement resolved with a third reviewer.

The following study characteristics were collected: name of the first author, year of publication of the study, country (origin) of data collection, sample size, study objective, age and gestational weight, associated pathologies, clinical parameters analyzed, moments of data evaluation, type/characteristic of the intervention, frequency and duration of therapy, statistical analysis and main results.

Methodological quality was analyzed by two evaluators, and any problem of divergence was resolved by consensus. The PEDro scale was used, based on the Delphi method, which aims to assist users in the methodological quality of clinical trials (criteria 2 to 9 of the scale) and statistical description (criteria 10 and 11 of the scale). The number of criteria met gave it its qualification. Item 1 was not calculated in the score, as it is an item that assesses the external validity of the study. Therefore, the score was between 0 and 10, characterizing the highest score as the best methodological quality.^22^

## RESULTS

**Figure 1.**
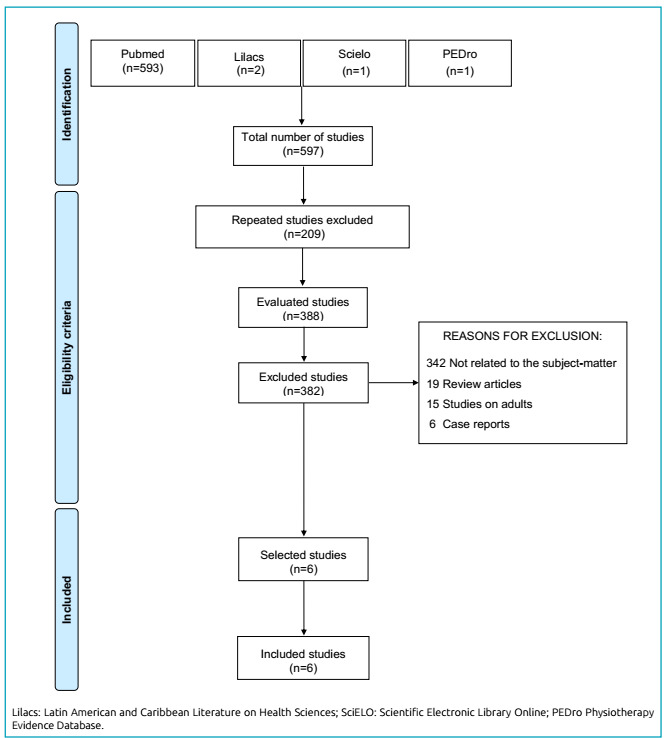
Systematization of the selection of studies in this review.

Of a total of 597 articles identified in the databases, only six were included in this review (Figure 1). Four studies (66.6%) were conducted in Brazil, and two (44.4%) were clinical, controlled and randomized trials (Table 1).

**Table 1:** Identification of the studies included in this review.

Authors	Country	Sample Size	Outline	Purpose of the study
Ribas et al.^23^	Brazil	26	RCT	Evaluate the effects of hammock positioning on reducing pain and improving sleep/wakefulness and vital signs
Jesus et al.^4^	Brazil	28	Quasi-experimental	Evaluate the effects of hammock positioning on behavioral status, pain and vital signs
Queiroz et al.^14^	Brazil	20	Quasi-experimental with cross-over	Verify the use of hammocks and prone positioning for pain relief and vital sign behavior
Costa et al.^8^	Brazil	30	Quasi-experimental with cross-over	Compare the effects of hammock positioning and the nest on the level of pain, posture and organizational status
Keller et al.^19^	Israel	20	RCT	Examine the effects of hammock positioning on growth, autonomic stability and neuromuscular maturity
Zanardo et al.^24^	Italy	15	Quasi-experimental	Evaluate the effects of hammock positioning on peripheral oxygen saturation

RCT: Randomized Controlled Trial

The samples, whose sample size varied between 15 and 30 participants, totaled 139 individuals. They contained neonates with a gestational age between 26 and 37 weeks and a gestational weight <2,240 g. Of these samples, two (44.4%) included PTNB with bronchopulmonary dysplasia and/or respiratory distress syndrome. The most investigated variables were vital signs (heart rate - HR, respiratory rate - RR, peripheral oxygen saturation - SpO_2_) and pain, in four (66.6%) studies. Also, changes were recorded in relation to behavioral, organizational and maturational states, among others. Three articles (50.0%) recorded the variables before, throughout and immediately after the interventions (Table 2).

**Table 2:** Characteristics of the evaluated samples.

Authors	Age (weeks)	Birth weight (g)	Associated pathologies	Variables evaluated	Moment of evaluation
Ribas et al.^23^	30 to 37	1620±0.51*	None	Pain, HR, RR, SpO_2_ and sleep/wake	10 minutes before and after the intervention
Jesus et al.^4^	28 to 36	<1500	None	Pain, HR, RR, SpO_2_ and behavioral status	10 minutes before and throughout the intervention (2, 20, 40, 60 minutes) and after 10 minutes
Queiroz et al.^14^	32^+^	1932^$^	RDS and BPD	Pain, HR, RR, SpO_2_, SBP, DBP, MAP and body temperature	Before and after the intervention^#^
Costa et al.^8^	32 to 35	1400-1800	None	Pain, flexing posture and organizational status	After changing diapers^#^
Keller et al.^19^	26 to 30	<1500	None	HR, RR, weight gain and neuromuscular maturation	Before, during, and after the intervention^#^
Zanardo et al.^24^	27 to 30	970-2240	BPD	SpO_2_	15 minutes before, during, and after the intervention

^+^Average gestational age of the evaluated group; *average weight of hammock positioning group; ^$^average gestational weight of the evaluated group; RDS: respiratory distress syndrome; BPD: bronchopulmonary dysplasia; HR: heart rate; RR: respiratory rate; SpO_2_: peripheral oxygen saturation; SBP: systolic blood pressure; DBP: diastolic blood pressure; MAP: mean arterial pressure; ^#^study did not specify the exact measurement time.

The majority (66.6%) of the studies placed the newborn in a supine position in the hammock positioning, while another portion (44.4%) put them in lateral decubitus. Regarding the studies that obtained a control group, two of the patients were placed in prone (nest) and two others in lateral decubitus in the nest. The duration of the intervention time varied between 15 and 180 minutes, and the interventions were performed in just one moment or in up to 10 days.

Among the main results observed, the improvement of vital signs (HR and RR) and pain in 75% of them (3/4 studies) and the increase in SpO_2_ by 50% (2/4 studies) stand out. There were gains in behavioral, organizational and maturational states, however the intervention with hammock positioning did not change weight gain, body temperature or blood pressure levels. Only one study (16.6%) reported worsening of SpO_2_ (Table 3).

**Table 3:** Main results of studies included in this review.

Authors	Type of intervention	Frequency	Duration	Statistical analysis	Pain	HR	RR	SpO_2_	Other outcomes
Ribas CG et al.^23^	Hammock positioning in LD Nesting position in LD	5 days	120 minutes	Intergroups	↓	↓	↓	↑	↑ Sleep and wake
Jesus VR et al.^4^	Hammock positioning in supine	1 moment	60 minutes	Intra-groups	↔	↓	↓	↔	↑Behavioral status
Queiroz CMB et al.^14^	Hammock positioning in RLD Nesting position in RLD	1 moment	40 minutes	Intra-groups	↓ ↓	↔ ↔	↔ ↔	↑ ↑	↔ SPB, DBP, MAP ↔ Body temperature ↔ SPB, DBP, MAP ↔ Body temperature
Costa KSF et al.^8^	Hammock positioning in supine Nest positioning in prone	1 moment	40 minutes	Intergroups	↓	-	-	-	↑ Flexor posture ↑ Organizational status
Keller et al.^19^	Hammock positioning in supine Nest positioning in prone	10 days	180 minutes	Intergroups	-	↓	↓	-	↑ Neuromuscular maturation ↔ Body weight
Zanardo V et al.^24^	Hammock positioning in supine	1 moment	15 minutes	Intra-groups	-	-	-	↓	-

LD: lateral decubitus; RLD: right lateral decubitus; intragroups: comparison of results before and after the intervention within the group; intergroups: comparison of intervention results between different groups; HR: heart rate (beats per minute); RR: respiratory rate (breath per minute); SpO_2_: peripheral oxygen saturation; ↑: increase; ↓: reduction; ↔: no change; -: not evaluated; SBP: systolic blood pressure; DBP: diastolic blood pressure; MAP: mean arterial pressure;

Finally, the average methodological quality was 5.33, varying between 3 and 8 points. Only two studies (33.3%) had scores >7, while the others (66.6%) were classified as having low methodological quality. The documents lost points mainly in the items about the hidden/random allocation, the blind assessors/therapists, and the homogeneity of the investigated groups (Table 4).

**Table 4:** Evaluation of the methodological quality of the studies included in this review.

Evaluated criteria	Ribas CG et al.^23^	Jesus VR et al.^4^	Queiroz CMB et al.^14^	Costa KSF et al.^8^	Keller et al. ^19^	Zanardo V et al.^24^
Eligibility criteria*	+	+	+	+	+	+
Random allocation	+	-	-	-	+	-
Hidden allocation	-	-	-	-	-	-
Similar groups	+	-	-	-	+	-
Blind participants	+	+	+	+	+	+
Blind therapists	-	-	-	-	-	-
Blind evaluators	+	-	-	-	-	-
Adequate follow-up	+	+	+	+	+	+
Intention to treat analysis	+	+	+	+	+	-
Between group comparisons	+	-	+	+	+	-
Point estimates and variability	+	+	+	+	+	+
Total score	8/10	4/10	5/10	5/10	7/10	3/10

*The item of the eligibility criteria does not contribute to the total score; + yes; - no.

## DISCUSSION

In this review, six studies were selected,^4^ ^,^ ^8^ ^,^ ^14^ ^,^ ^19^ ^,^ ^23^ ^,^ ^24^ that evaluated the effects of hammock positioning on some clinical parameters, such as pain and vital signs of PTNB admitted to the NICU. Although hammock positioning appears to cause improvement in HR, RR and pain levels,^4^ ^,^ ^8^ ^,^ ^14^ ^,^ ^23^ more randomized clinical trials are still needed to confirm these therapeutic findings. To date, the low methodological quality of the selected studies makes the results reported in this review inconsistent, limiting its recommendation in professional clinical practice.

Hammock positioning is a humanization strategy within the NICU, based on the synchronous-active theory of the newborn's neurobehavioral organization, considering that it aims to optimize a child's interaction with their neurobehavioral environment and subsystems.^2^ Our findings showed that almost all of the studies^4^ ^,^ ^23^ ^,^ ^8^ observed positive effects of the hammock on vital parameters, including HR and RR. According to some authors,^14^ ^,^ ^23^ this intervention simulates the intrauterine environment through physiological positioning and the small swing generated by this device from the help of the newborn's body and respiratory movements. Physiologically, this could have a positive impact on the autonomous system, regulating respiratory movements and heart rate.^25^

Premature babies have immature inhibitory pathways that come from pain, due to the incomplete maturation of their central nervous system. A recent study highlighted that PTNB feel pain during invasive procedures in the NICU. Aspiration through an orotracheal tube and/or an airway is the main reason for this pain.^26^ Furthermore, the routine procedures and painful stimuli within the unit increasingly corroborate the need for minimal handling of these patients.^27^ Non-pharmacological and low-risk resources that promote the reduction of stress and pain levels in PTNB should be encouraged, such as hammock positioning.^4^ In this review, three studies^8^ ^,^ ^14^ ^,^ ^23^ reported that such intervention generated a reduction in pain levels, by using different assessment tools.^8^ ^,^ ^14^ ^,^ ^23^ In general, these scales assess the behavioral, physiological and contextual aspects of the occurrence of pain.^8^ ^,^ ^14^ ^,^ ^23^ Only the research by Jesus et al.^4^ showed no change in the levels of this outcome. This result can be attributed to the fact that the studied group consisted of PTNB with greater immaturity of the neural pathways of pain, and a lower gestational age (28 to 36 weeks) and birth weight (<1,500 g) was considered in their sample.

Previous studies have shown that different positions positively influence PTNBs, contributing to lower energy expenditure, improved oxygenation, reduced episodes of gastroesophageal reflux and lower thoracoabdominal asynchronism.^28^ ^,^ ^29^ Many devices, both artisanal and commercial, are available to assist in this positioning, favoring body flexion, including nest and hammock positioning, a fact documented here.^30^ Although therapeutic positioning is considered a to be a common management practice within the NICU, it can alternate in decubitus, depending on the location, the therapeutic resource and the underlying pathology. Scientifically, the prone position is documented as being more beneficial compared to the supine position. This occurs due to greater stability of the rib cage and more space for the diaphragmatic muscle fiber, which enhances its action. However, as the results of the present study are directed to the use of hammocks, no study used this type of positioning.

Although therapeutic positioning has a positive relationship with SpO_2_, only two studies reported significant changes in this variable with the use of hammock positioning, while two others reported not changing^4^ or clinical worsening.^24^ The lack of benefits in these two studies^4^ ^,^ ^24^ could be explained, at least in part, by the greater severity of the samples, including the very/extremely low weight,^4^ ^,^ ^24^ hemodynamic instability^24^ and bronchopulmonary dysplasia.^24^ Some studies carried out in children and adults have shown greater benefits in saturation and partial oxygen pressure in the prone position compared to supine, with this improvement being more evident in the presence of lung disease.^29^ ^,^ ^31^ ^,^ ^32^ ^,^ ^33^ This could explain the fact of the worsening treatment of PTNB in the study by Zanardo et al.^24^, as the patients were positioned in supine in this intervention. Furthermore, it is known that a ventilatory strategy to minimize lung injury in bronchopulmonary dysplasia is the use of the prone position, which is different from the position used by its sample.^31^ It is recommended that this type of therapy be evaluated and used by trained professionals. Furthermore, it should not be applied indiscriminately, without monitoring, and preferably by physiotherapists.^9^

 In health care practice, the presence of pain and discomfort causes changes in the blood pressure of PTNB due to the different regulatory mechanisms. Among them are the neural mechanisms linked to the autonomic nervous system and the hormonal system, which is related to renin-angiotensin.^34^ ^,^ ^35^ However, in this review, only one study^14^ reported that there were no significant changes in blood pressure or body temperature with the use of hammock positioning. Although it is not justified in the scientific literature, it seems that hammocks when applied only once and for a short period of time do not impact these parameters, unlike the outcomes HR, RR and pain.^4^ ^,^ ^8^ ^,^ ^23^ However these clinical markers can be modified with other resources, such as, for example, a heated cradle, antipyretic and blood pressure regulating medications, among others.^36^ ^,^ ^37^ ^,^ ^38^ ^,^ ^39^

The present study had some limitations. One of them was the fact that we included PTNB with associated diseases, in addition to prematurity.^14^ ^,^ ^24^ We chose to leave them in the sample because the majority of patients admitted to the NICU have other comorbidities in clinical practice.^40^ In addition, the effects investigated in this study reflect more clinical changes in the short term (60 minutes),^4^ ^,^ ^8^ ^,^ ^14^ ^,^ ^24^ since most studies evaluated the effects in just one period of time.^4^ ^,^ ^8^ ^,^ ^14^ ^,^ ^24^ The low methodological quality of the research included in this review constitutes the greatest restriction of the study. Only two studies^19^ ^,^ ^23^ obtained a methodological quality ≥7 points on the PEDro scale. The absence of hidden allocation,^4^ ^,^ ^8^ ^,^ ^14^ ^,^ ^19^ ^,^ ^23^ ^,^ ^24^ blinding therapists ^4^ ^,^ ^8^ ^,^ ^14^ ^,^ ^19^ ^,^ ^23^ ^,^ ^24^ and evaluators^4^ ^,^ ^8^ ^,^ ^14^ ^,^ ^19^ ^,^ ^24^ and the lack of a control group^4^ ^,^ ^24^ makes the results reported here fragile.

In conclusion, although hammock positioning seems to cause improvement in some clinical parameters, mainly HR, RR and pain in PTNB, the low methodological quality of the selected studies makes the results reported here inconsistent. Thus, new randomized clinical trials are needed to confirm these therapeutic findings, in order to assess, in the future, whether there is sufficient evidence to recommend this method in the NICU.
